# The clinical effects of laser preparation of tooth surfaces for fissure sealants placement: a systematic review and meta-analysis

**DOI:** 10.1186/s12903-019-0892-4

**Published:** 2019-09-02

**Authors:** Yunhan Zhang, Yan Wang, Yandi Chen, Yang Chen, Qiong Zhang, Jing Zou

**Affiliations:** State Key Laboratory of Oral Diseases & National Clinical Research Center for Oral Diseases & Department of Pediatric Dentistry, West China Hospital of Stomatology, Sichuan University, Chengdu, 610041 China

**Keywords:** Lasers, Pit-and-fissure sealants, Enamel preparation, Systematic review, Meta-analysis

## Abstract

**Background:**

This systematic review aimed to assess the clinical effects of laser preparation compared to other types of chemical or mechanical preparation of tooth surfaces used in fissure sealant placement.

**Methods:**

A systematic literature search was conducted up to January 2019, through Pubmed, Scopus, Medline/EMBASE via OVID and the Cochrane library. Only randomized clinical trials were included.

**Results:**

Five studies were included in the systematic review and three were included in the meta-analysis. All the studies used acid-etching as a comparator to lasers. All the included studies were rated as having an overall high risk of bias introduced by performance bias. Three studies assessed the clinical effects of fissure sealants placed by acid or laser etching, one compared acid etching versus laser combined with acid etching and one investigated the influence of lasers on the objective and subjective parameters of stress during sealant application in children. The meta-analysis showed no significant difference between laser preparation and conventional acid-etching preparation at 3- (*P* = 0.08), 6- (*P* = 0.49), and 12-month (*P* = 0.87) follow-ups. One study reported that laser preparation as an adjunct to acid-etching enhanced the retention rate. No significant difference in the incidence of caries was reported. And no significant differences were found in heart rates, oxygen saturation or degree of the patient dental anxiety between acid-etching and laser preparation.

**Conclusion:**

The present limited evidence suggests that lasers could be an effective pretreatment method. The retention rate was similar to that of conventional acid etching. However, the included studies had an overall high risk of bias and more rigorously designed research is needed.

## Background

In recent years, high attention has been paid to preventive dentistry, one goal of which is to prevent dental caries and reduce the risk of caries. Many techniques are available for the prevention and reduction of dental caries, including dietary modification, topical and systemic fluorides, sugar substitutes, pit-and-fissure sealants, caries vaccines, etc. Pit-and fissure-sealing is one of the most highly recommended and widely accepted preventive procedures, especially for preventing caries in newly-erupted permanent molars [1–4]. Studies have reported that more than two-thirds of caries in children develop on occlusal surfaces [5, 6]. Occlusal surfaces have pits and fissures, which are highly susceptible to caries because they accumulate bacteria more easily and plaque is difficult to remove from the surfaces [7, 8]. A fissure sealant is a dental material that is placed in the pits and fissures of teeth to prevent the entrance of cariogenic bacteria and their nutrients inside these anatomical fissures [9]. It was first introduced to protect occlusal pits and fissures for dental caries by Cueto and Buonocore in 1967 [10].

The effectiveness of pit-and-fissure sealing to prevent dental decay is closely related to the retention of the sealants. Roughing the enamel surface to promote the adhesion of fissure sealants is one of the most important ways to increase the retention rate [11]. Acid-etching is a well-accepted, standard pretreatment technique for enamel surfaces to promote the adhesion of restorative materials. Nevertheless, the pellicle and remaining debris might not be removed from the base of the fissures with conventional prophylaxis and etching procedures. Furthermore, saliva contamination after the etching procedures can also compromise adhesion [12]. Therefore, alternative methods to acid etching, such as enameloplasty, an air-polishing system and laser treatment have been proposed for preparing fissures for sealant retention [13, 14].

Laser application in dentistry has been a research interest for the past 30 years and has recently risen in popularity. Laser irradiation of hard dental tissue modifies the calcium: phosphorus ratio, reduces the carbonate: phosphate ratio and leads to the formation of more stable and less acid-soluble compounds, thus reducing the susceptibility to acid attacks and caries [15–17]. In addition, laser treatment can promote sterilization of the fissures because of its action on dental plaque. Therefore, the use of lasers has been suggested as a pretreatment method to roughen enamel.

Studies on the quality of lasers as a preparation method for the enamel surface before sealant application, however, are inconclusive. A review and meta-analysis of the literature based on in vitro experiments showed that pretreatment with phosphoric acid led to lower microleakage in occlusal sealants than erbium:yttrium-aluminum-garnet lasers (Er: YAG) and air abrasion [18]. Besides, as a clinical technology, the effect of laser preparation on patients’ subjective experiences was still unclear. This study was conducted to assess the clinical effects of laser preparation compared to other types of chemical or mechanical preparation of the tooth surfaces used in fissure sealant placement.

## Methods

### Focused question

This systematic review was reported in accordance with recommendations of the Preferred Reporting Items for Systematic reviews and Meta-Analyses (PRISMA) statement [19]. The research aimed to systematically retrieve and analyze clinical studies assessing the effects of laser preparation compared to other tooth surface pretreatment methods used in fissure sealant placement. The PICO principle, detailed below, was applied during the assessment.

Participants: patients who were caries-free and had untreated premolars and/or molars and/or primary molars suitable for pit-and fissure-sealing.

Interventions: use of lasers as a pretreatment method for pit-and-fissure sealing.

Comparators: use of any other mechanical or chemical preparation for pit-and-fissure sealing, such as acid-etching, enameloplasty or air abrasion.

Outcomes: the retention rate of fissure sealants, the incidence of dental caries, adverse events, clinical time, patient acceptability and anxiety.

### Selection criteria

The inclusion criteria were: 1) randomized clinical trials; 2) studies using laser and the other pretreatment methods, including phosphoric acid and/or enameloplasty and/or air abrasion to roughen the enamel before sealant application; 3) studies reporting details of the materials and methods, such as tooth preparation designs, isolation methods, materials of the sealants and parameters of the lasers; 5) studies with quantitative clinical outcomes, such as retention rates or the incidence of caries. The exclusion criteria were 1) in vitro or animal studies, case reports, and case series, review articles, or opinion articles; and 2) studies published in other languages besides English.

### Search strategy

The following databases were searched from inception to January 2019: Pubmed, Scopus, Medline/EMBASE via OVID and the Cochrane library. The following search terms are used in Pubmed: (“lasers” [MeSH Terms] OR “lasers” [All Fields] OR “laser” [All Fields]) AND “pit” [All Fields] AND (“pit and fissure sealants” [MeSH Terms] OR (“pit” [All Fields] AND “fissure” [All Fields] AND “sealants” [All Fields]) OR “pit and fissure sealants” [All Fields] OR (“fissure” [All Fields] AND “sealant” [All Fields]) OR “fissure sealant” [All Fields]).

ClinicalTrials.gov (www.clinicaltrials.gov) and the International Standard Randomised Controlled Trial Number (ISRCTN) registry (www.isrctn.com) were screened to identify unpublished and ongoing trials. Grey literature was also investigated through OpenGrey. In addition, the reference lists of the included studies were manually searched to find any potentially eligible study.

### Study selection

The titles, abstracts and full-texts of potentially relevant studies were read and assessed by two authors (Y.H.Z and Y.W) to judge whether they met the inclusion and exclusion criteria. Unrelated studies were initially excluded based on the titles and abstracts and any conflict was solved by a third author (J.Z).

### Data extraction

The characteristics and numerical data in the included studies were extracted independently by two authors (YHZ and YW) using a prepared data collection form. Any disagreement was solved via discussion or by a third author (JZ). The data obtained included follow-up duration, the number of patients, quantity of the sealants, patient age range, drop-out rate of the subjects, the preparation protocol, the laser parameters, the type of sealant, tooth location, retention rate, the incidence of caries, patient acceptance, time consumption and dental anxiety. Retention in this systematic review was defined as completely retained [20].

### Assessment of risk of bias in the included studies

The risk of bias in the RCTs was assessed according to the Cochrane Handbook for Systematic Reviews of Interventions [21]. Two review authors (YHZ and YW) independently assessed and scored the studies to identify any potential sources of systematic bias. The related risk of bias for each domain was rated at three levels: low risk, high risk, or unclear risk. The comprehensive methodological quality of a study was classified as low risk of bias (six domains assessed as low risk), moderate risk of bias (one or more domains assessed as unclear risk) or high risk of bias (one or more domains assessed as high risk). Disagreements between the authors were resolved through discussion and, if needed, by consultation with another author. Additional risk of bias tables for the included studies are presented.

### Statistical analyses

Statistical heterogeneity was assessed using a Chi-squared test and the Higgins index (I^2^). Heterogeneity was considered statistically significant for *P* < 0.1. Meta-analyses were performed when there was little clinical heterogeneity. A random-effects model was used for I^2^ > 50% and a fixed-effect model was used for I^2^ ≤ 50%. Odds ratios (OR) and their corresponding 95% confidence intervals (CI) were calculated. The statistical significance of the hypothesis test was set at a α = 0.05 (two-tailed z tests) All analyses were performed using Revman 5.3 software. The data were summarized qualitatively when a meta-analysis could not be performed.

## Results

### Search results

**Fig. 1:**
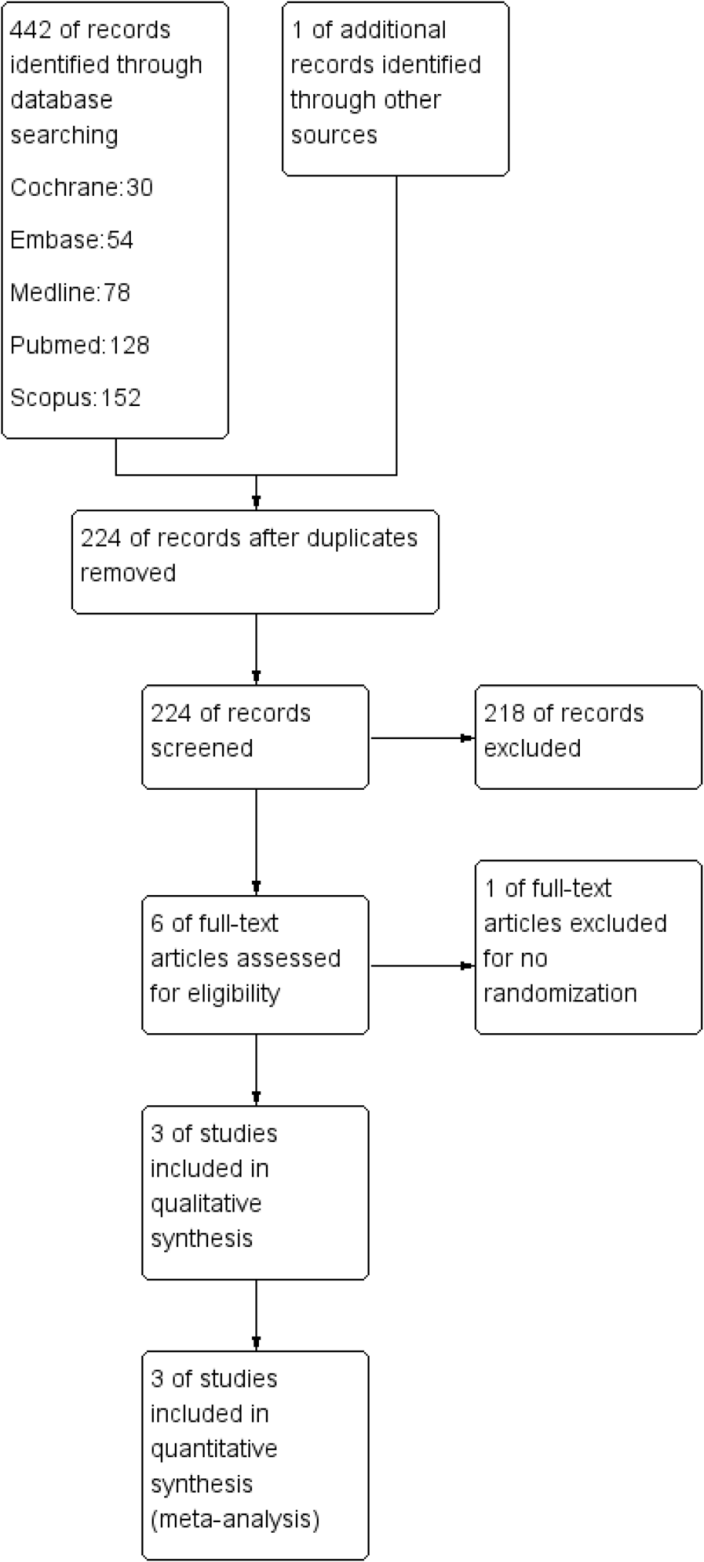
Systematic review study selection flow chart

The search strategy is outlined by a flow diagram and presented in Fig. 1. Initially, 442 published studies were identified through the electronic search and one additional record from other sources. After the removal of duplicates, 224 studies remained. Another 218 articles were removed after analysis of the titles and abstracts, leaving six studies. After evaluating the full texts of the articles in detail, five studies remained eligible for this systematic review. Finally, three studies were included in the meta-analysis.

There were no ongoing studies or grey literature. There was a completed study “The effect of Er:YAG laser on clinical success of a hydrophilic fissure sealant” (NCT03718689) that met the inclusion criteria of this systematic review, retrieved from ClinicalTrials.gov. We contacted the study director for further information but she refused to supply data. After the electronic searches, the references of the included studies were hand-searched, however, no further applicable studies were found.

### Characteristics of the included studies

Study characteristics and results of the included studies are summarized and presented in Table 1. The included studies were conducted in four countries in subject numbers ranging from 16 to 64. The participants’ ages ranged from six to 38 years old. Follow-up duration ranged from 12 to 36 months, except for one study without a follow-up [26]. Two studies reported drop-out rates according to the follow-up periods [23, 25]. One reported a 17.65% drop-out rate [22] and two reported 0% [24, 26]. Three studies assessed the clinical performance of fissure sealants placed by acid or laser etching and one compared acid etching versus laser combined with acid etching. Four of the studies assessed retention rates [22–25], two reported the incidence of caries [22, 24] and one evaluated patient acceptance [23]. The study by Shindova et al. evaluated heart rate, oxygen saturation and the subject’s degree of dental anxiety before and after the dental visit [26].

**Table 1:** Study characteristics and results of the included studies

Study	Country	Design	Follow-up duration (months)	Number of patients (drop out)	Age range (years)	Tooth Location	Preparation method	Isolation	Sealant	Outcome variables	*P* value
Durmas 2017 [22]	Turkey	RCT, split-mouth	18	51 (9)	7–10	T: #26, #36 C: #16, #46	T: acid-etch+Er: YAG laser C: acid-etch	Cotton rolls	Grandio Seal (VOCO)	Retention rate, incidence of caries	Retention rate: 3mo *P* = 0.49; 6 mo *P* = 0.31; 12 mo *P* = 0.01^*^; 18mo *P* = 0.02^*^; Incidence of caries: *P* > 0.05
Kumar 2016 [23]	India	RCT, split-mouth	12	50 (3mo = 5; 6mo = 6; 9mo = 7; 12mo = 11)	6–12	#16, #26, #36, #46	T: Er, Cr: YSGG laser C: acid-etch	Rubber dam	Clinpro Sealant (3 M ESPE)	Retention rate, patient acceptability	Retention rate: 3mo *P* = 0.12; 6mo *P* = 0.76; 9mo *P* = 0.85; 12mo *P* = 0.78 Patient acceptability: *P* = 1.000
Karaman 2013 [24]	Turkey	RCT, parallel design	24	16 (0)	20–23	Permanent premolars and molars	T: Er, Cr: YSGG laser C: acid-etch	Cotton rolls	Clinpro Sealant (3 M ESPE)	Retention rate, incidence of caries	Retention rate: 6mo *P*>0.05; 12mo *P*>0.05; 18mo *P*>0.05; 24mo *P*>0.05
Walsh 1996 [25]	Australia	RCT, split-mouth	36	20(12mo = 2; 18mo = 12;36mo = 19)	15–38	Permanent premolars and molars	T: carbon dioxide laser C: acid-etch	Cotton rolls	Delton Clear (Johon& Johnson Dental Care Company)	Retention rate	3mo NR; 6mo NR; 12mo *P* = 0.41
Shindova 2018 [26]	Bulgaria	RCT, parallel design	–	64 (0)	6–12	Intact teeth with no decay on the occlusal surface	T: Er: YAG laser+acid-etch C: acid-etch	Cotton rolls	Pit and fissure sealant (DMP Ltd)	Dental anxiety	*P*>0.05

*mo* month, *RCT* Randomized comparison trial, *Er: YAG* Erbium:yttrium-aluminum-garnet, *Er, Cr: YSGG* Erbium, chromium: yttrium-scandium-galium-garnet, *NR* Not reported, *FS* Fissure sealants, * statistically significant, *T* Test group, *C* Comprison group

In terms of the methodological characteristics (Table 1), three of the included studies adopted a split-mouth design [22, 23, 25]. All of them used acid-etching as the only comparator. Out of the five studies, two used erbium, chromium: yttrium-scandium-galium-garnet (Er, Cr: YSGG) lasers alone in the intervention group [23, 24], one used carbon dioxide laser [25] and two studies used Er: YAG laser combined with acid etching [22]. Four studies isolated teeth from the oral environment with cotton rolls [22, 24–26] and the remaining one used a rubber dam [23]. Two studies performed pit-and-fissure sealants on the first permanent molars [22, 23], two were performed on the permanent premolars and molars [24, 25] and one was performed on any intact teeth without caries on the occlusal surface [26].

### Laser parameters of the included studies

The laser parameters of the included studies are presented in Table 2. One study used a carbon dioxide laser [25], two studies used Er: YAG [22, 26] and two used Er, Cr: YSGG [23, 24]. The power of the carbon dioxide laser was 5 W and that of the erbium lasers ranged from 0.7 W to 2.0 W. Two studies reported that the exposure time depended on the time needed to guide the laser beam evenly across the pits and fissures to be irradiated [22, 24], one did not report exposure time [26] and the exposure time in the remaining study ranged from 7 to 10 s [23, 25]. One study reported that the energy density was 67 J/cm^2^ [26] and one reported a power density of 530.5 W/cm^2^ [23]. The laser application methods were similar, presenting small differences in the tip-to-tissue distance, tip diameters and angles. The carbon dioxide system used by Walsh was not water-cooled [25], while the erbium laser systems used by other researchers were equipped with water-cooled systems [22–24, 26].

**Table 2:** Laser parameters of the included studies

Study	Type of laser	Mode	Wavelength	Power/Frequency	Power/Energy density	Time of exposure	Method of application
Durmus 2017 [22]	Er: YAG laser	Pulse wave	2.94 μm	2 W/10 Hz	NR	The duration of exposure depended on the time needed to guide the laser beam evenly across the pits and fissures to be irradiated	The laser beam was aligned perpendicular to the fissure in noncontact mode at a distance of 1 to 2 mm in accordance with the manufacturer’s instructions for etching, and a beam spot size is 0.6 mm.
Kumar 2016 [23]	Er, Cr: YSGG laser	Pulse wave	2.78 μm	1.25 W/20 Hz	530.5 W/cm^2^	10s	The treatment was performed with a 600 μm diameter tip aligned perpendicularly to the target area at a distance of 1–2 mm from the surface.
Karaman 2013 [24]	Er, Cr: YSGG laser	Pulse wave	2.78 μm	1.25 W/10 Hz	NR	The duration of exposure depended on the time needed to guide the laser beam evenly across the pits and fissures to be irradiated	The treatment was performed with a 600 μm diameter tip aligned perpendicularly to the target area at a distance of 1–2 mm from the surface.
Walsh 1996 [25]	Carbon dioxide lase	Pulse wave	NR	5 W/20 Hz	NR	Maximum total time for any one tooth is 7 s	The laser energy was delivered using a flexible waveguide fitted with an angled (105°) handpiece and an 0.8 mm diameter ceramic tip.
Shindova 2018 [26]	Er: YAG laser	Pulse wave	2.94 μm	0.7 W/10HZ	67 J/cm^2^	NR	tip-to-tissue distance was non-contact mode (1.5 mm) and tip diameter was 600 μm

*RCT* Randomized comparison trial, *Er: YAG* Erbium:yttrium-aluminum-garnet, *Er, Cr: YSGG* Erbium, chromium: yttrium-scandium-galium-garnet, *NR* Not reported

### Risk of bias

**Fig. 2:**
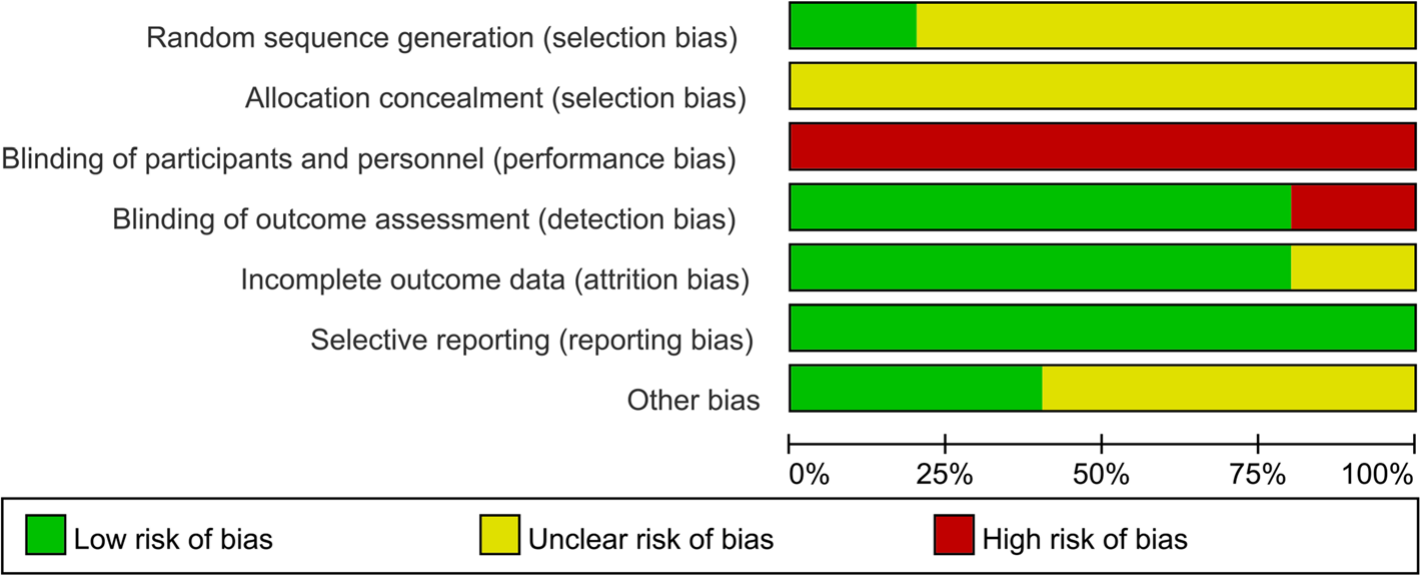
Risk of bias graph. The review authors’ risk of bias assignments presented as percentages across all included studies

**Fig. 3:**
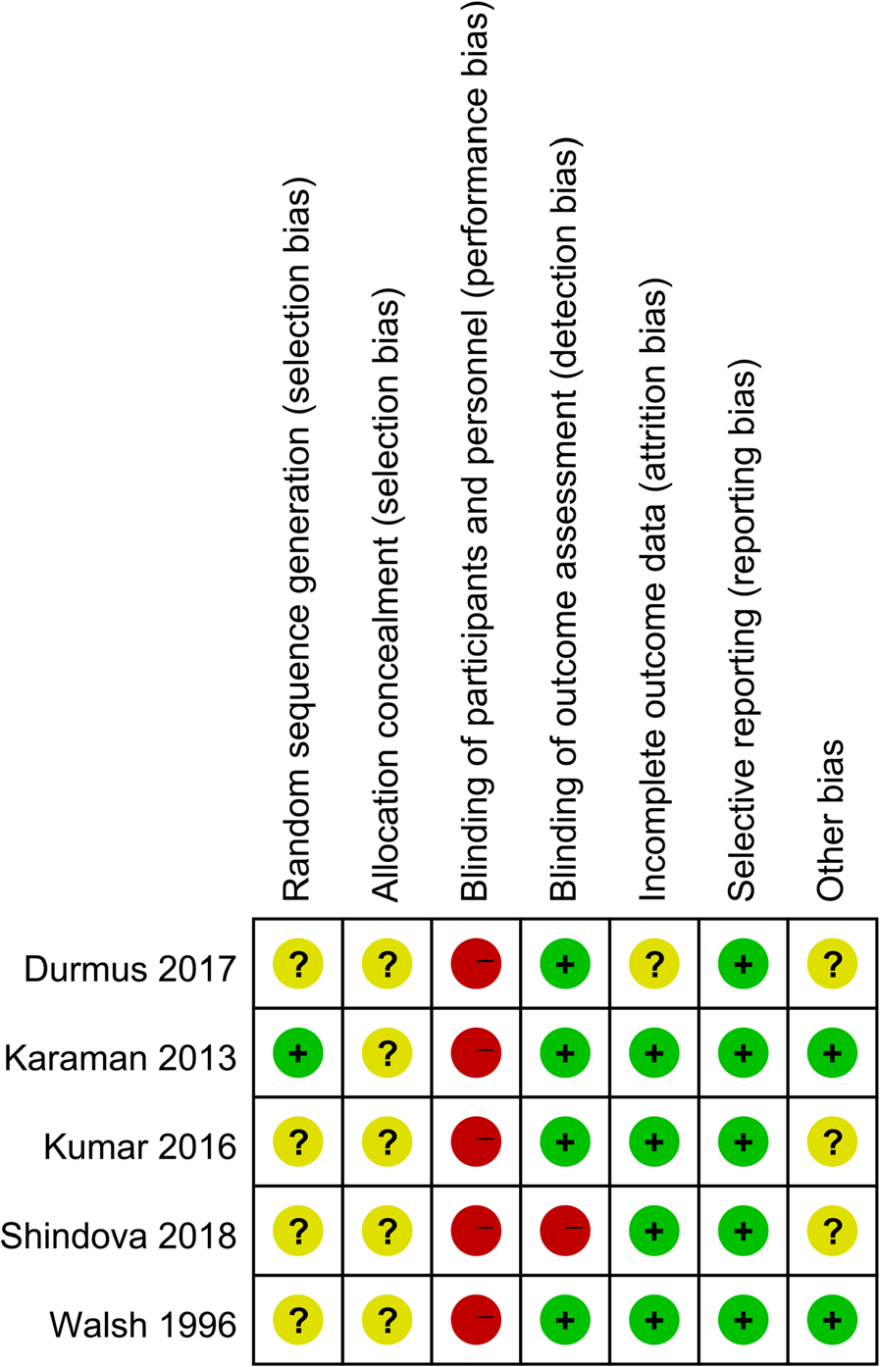
Risk of bias summary. The review authors’ risk of bias assignments for each included study

The risk of bias for the included studies is summarized in Fig. 2 and given in detail in Fig. 3. All the studies had an overall high risk of bias. Because of the enormous difference in instruments and usage, all the studies included presented inevitable high risk in blinding of the intervention operators. Four studies used single-blind designs [22–25], and the remaining study evaluated the patients’ subjective outcomes and was assessed as high risk in detection bias [26]. Only Karaman reported the use of a random number table to generate random sequences, while the other studies did not refer to allocation concealment [24]. The included studies were considered to be low risk of bias in selective reporting. Four studies showed low risk in attrition bias [23–26] and the study by Dumurs did not explain the unavailable data [22]. Two studies showed low risk of other bias [24, 25] and the remaining were unclear (Figs. 2 and 3).

### Outcomes variables

#### Retention rate

**Fig. 4:**
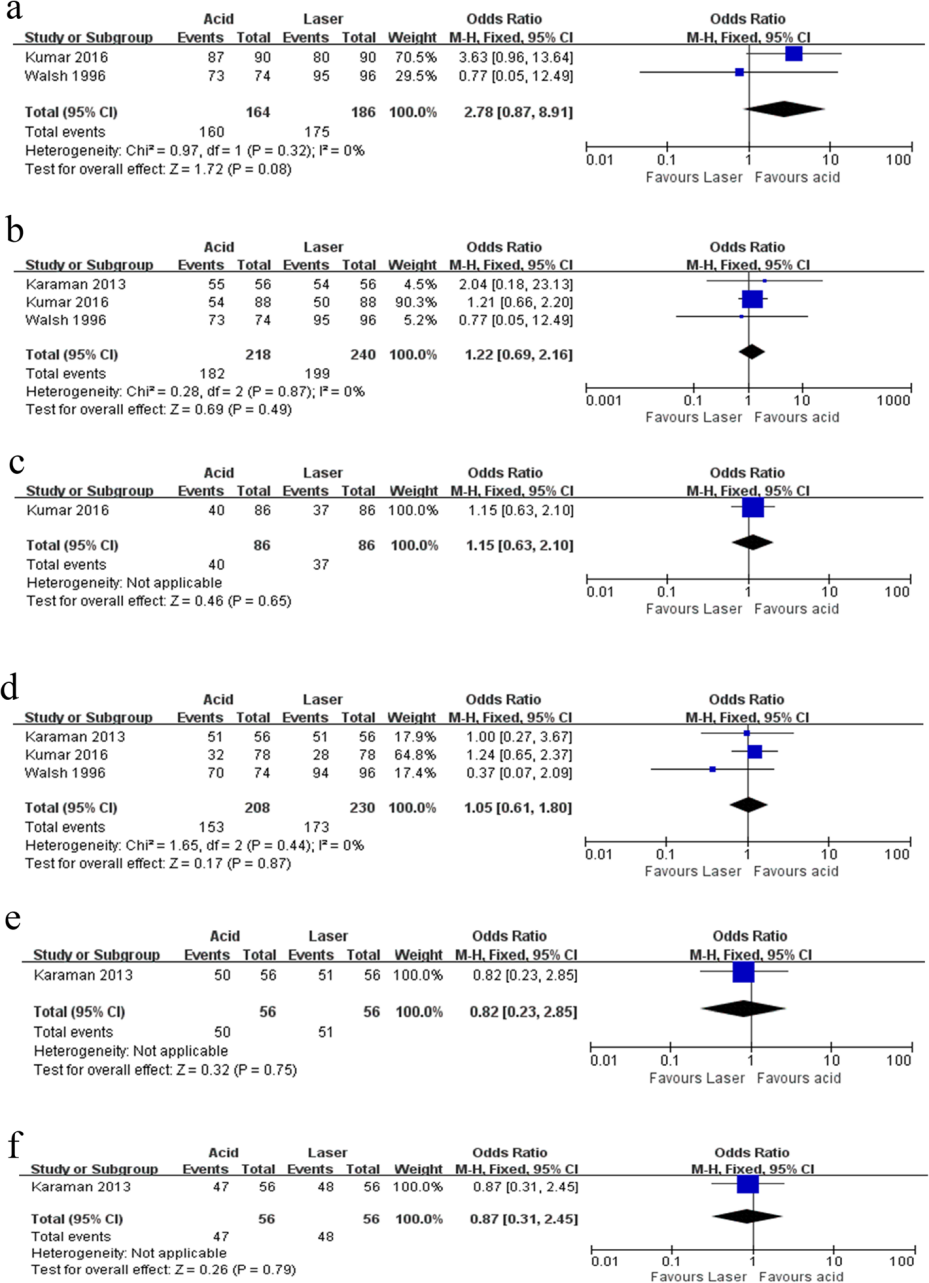
Forest plot of the retention rates in acid etching and laser preparation. **a**. 3 months, **b**. 6 months, **c**. 9 months, **d**. 12 months, **e**. 18 months and **f**. 24 months

Four of the included studies used Simonsen’s criteria to evaluate the retention rate, which fell into three categories: completely retained, partially retained and completely lost [20, 22–25]. Retention rates reflected completely retained sealants. The retention rates were reported according to the different follow-up times in all the studies, ranging from 3 months to 24 months. At the end of the follow-up periods, the retention rates ranged from 35.9 to 97.92% in the laser only group, 77.4% in the laser combined with acid etching group and 41.0 to 94.59% in the acid-etching only group (Table 1). Three studies used lasers alone in the test group [23–25], enabling synthesis of the data by meta-analysis. We conducted a meta-analysis for studies that evaluated retention at the same time after sealant application to get more reliable results. It is worth noting that we only adopted the retention rate at 12 months due to the high number of patients lost to follow-up at 18 months (12 subjects dropped-out out of 20) and 30 months (19 subjects dropped-out out of 20) in Walsh’s study. Figure 4 shows the Forest plot for the retention rates in acid-etching and laser preparation, including Er, Cr: YSGG and CO_2_ lasers. The fixed-effect model was used in this analysis. The odds ratio was 2.78 at the 3-month follow-up (95% CI: 0.87–8.91; *P* = 0.08), suggesting that there was no significant difference between acid-etching and laser preparations. The heterogeneity between studies was 0% (I^2^). This result was similar to those obtained at the 6- and 12-month follow-ups. The odds ratios were 1.22 (95%CI: 0.69–2.16; *P* = 0.49) and 1.05 (95%CI: 0.61–1.80; *P* = 0.87), respectively. No subgroup analysis was performed due to the small number of included studies (Fig. 4).

**Fig. 5:**
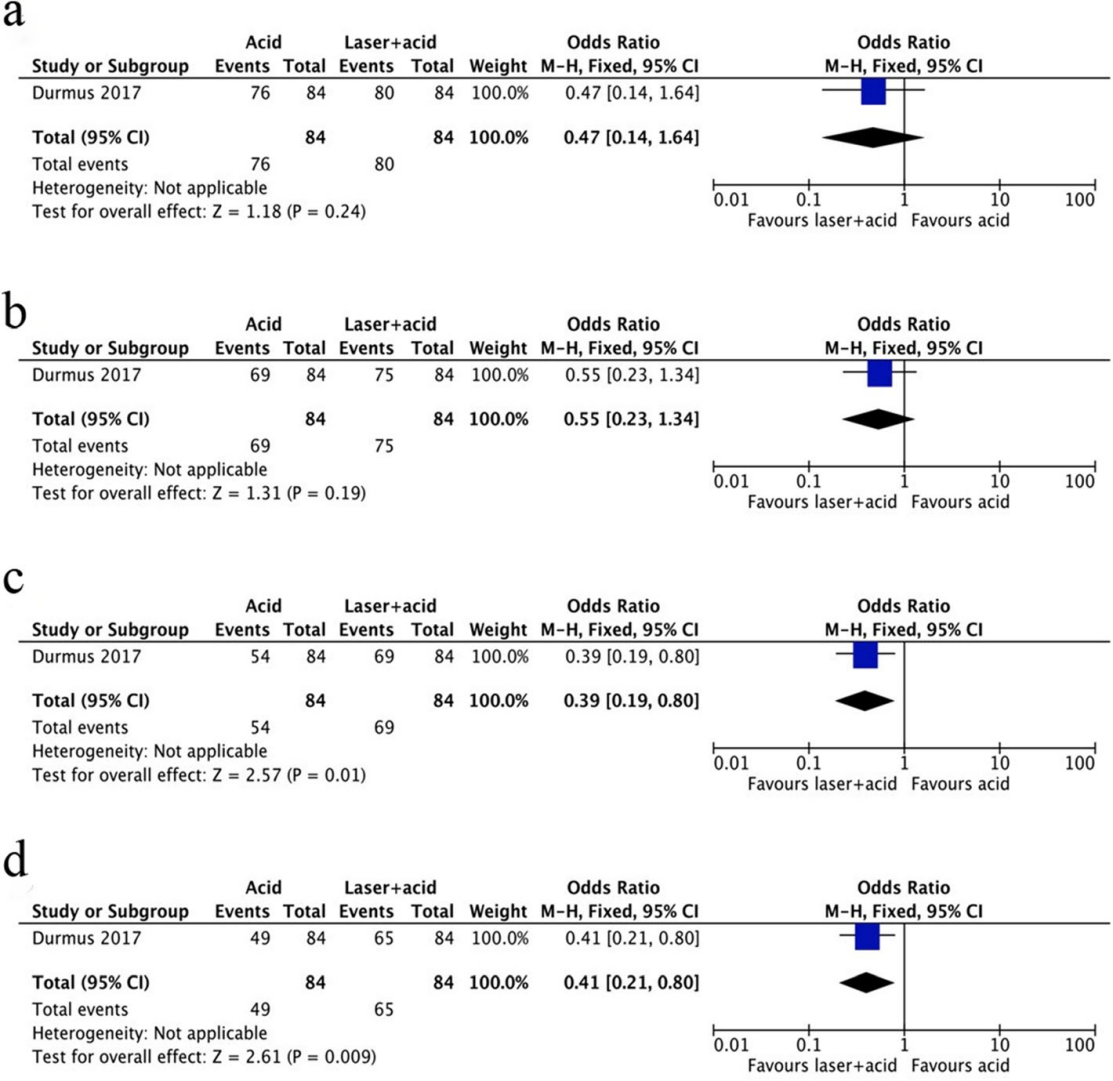
Forest plot of the retention rates in acid etching and laser preparation plus acid etching. **a**. 3 months, **b**. 6 months, **c**. 12 months and **d**. 18 months

One other study used Er: YAG laser combined with acid etching in the intervention group [22]. The sealant retention rate was significantly higher at 12 and 18 months compared to the acid-etched group, while there were no significant differences in retention rates between these two preparation methods at the 3- and 6-month follow-ups (Table 1). The Forest plot presented a visual representation of this outcome, in which the OR was 0.47 (*P* = 0.24) at the 3-month follow-up, 0.55 (*P* = 0.19) for the 6-month, 0.39 (*P* = 0.01) for the 12-month and 0.41 (*P* = 0.009) for the 18-month follow-up, respectively (Fig. 5).

#### Incidence of caries

Two studies reported the incidence of caries. In Karaman’s study, the incidence of caries in both the Er, Cr: YSGG laser group and the acid etching group was 0% [24]. Durmus reported that the incidence of caries was 10% in the Er: YAG laser plus acid etching group and 22% in the acid-etching group [22], however, the difference was not statistically significant.

#### Adverse events

No adverse events, such as pulpal sensation, post-lasing thermal sensitivity, pulpitis or other laser-induced pathology, were reported in the included studies.

#### Clinical time

Walsh et al. reported that the total clinical time of the laser conditioning procedure was shorter than the conventional acid preparation time (7 s versus 70 s) [25]. Kumar reported that the Er, Cr: YSGG laser exposure time was about 10 s, while the time to apply acid and rinse with water was more than 35 s in the acid-etching group [23]. In the other studies, the time spent on laser (Er:YAG and Er, Cr: YSGG) preparation was not described accurately and could not be calculated by the data provided in the articles. It is worth noting, however, that there was a significant difference in clinical time between Walsh’s carbon dioxide laser treatment and other erbium laser treatments. In Walsh’s study, the carbon dioxide laser treatment was performed under dry conditions. The security of pulpal safety was based on laboratory studies with relevant laser conditioning parameters. In contrast, all of the erbium laser systems used by other researchers had water-cooled systems to prevent the surface from overheating. This means it had to be air-dried for a few seconds like the acid-etching procedure but no studies recorded the time for this process. In the included studies, erbium lasers systems needed more clinical time than the carbon dioxide system used by Walsh.

#### Patient satisfaction

In terms of patient acceptance of the procedures, only Kumar and colleagues [23] used the visual analogue scale to score patient acceptance, which showed that both Er, Cr: YSGG laser and acid etching preparations were well accepted. In terms of dental anxiety, one included study subjectively and objectively evaluated dental anxiety when lasers were used to pretreat the dental surface in the process of pit-and-fissure sealing [26]. The authors did not find any significant differences between the initial and final subjective scores for dental anxiety between the Er: YAG laser combined with acid etching conditioning group and the acid etching group. They also did not find significant differences in physiological stress indicators, including heart rate and oxygen saturation, between these groups. Therefore, it was concluded that use of the Er:YAG lasers in preparation for sealant application did not provoke anxiety subjectively or objectively. Furthermore, it was well accepted by children in the dental environment [26].

## Discussion

The present systematic review included randomized clinical trials to evaluate retention rates, secondary caries, clinical times and the effect on patient psychology when lasers were used in preparation for sealant placement. A total of five studies were included in the systematic review and three of them were quantitatively analyzed. The assessment of quality exhibited overall high risk, indicating an under-grading of the quality of the existing evidence. In terms of retention rates, the available evidence suggested that laser preparation was not significantly different than acid-etching. Meta-analysis showed similar outcomes of laser and acid preparation after three, six, and 12 months. The incidence of secondary caries between laser preparation and acid preparation was similar. Furthermore, it was of highly accepted by patients and did not provoke dental anxiety.

Findings from this systematic review and meta-analysis indicated the possibility and reliability of laser preparation as a substitute for phosphoric acid etching before sealant placement, indicated by its similar retention and secondary caries rates, as well as high psychological acceptance by the patients. However, the results and conclusions should be interpreted carefully due to differences in study methodology and laser parameters.

### Effects of study methodology on outcome

A few methodological differences are worth noting. First, regarding the outcomes of the present studies, split-mouth design, in which the effects of inter-subject variation can be minimized when the individual is self-matched or self-controlled, is more suitable than parallel design for clinical trials conducted in the oral cavity [27, 28].

Caries risk may influence the retention rate and the incidence of caries. In high-risk children, the enamel of the occlusal pit and fissures has already undergone tissue structural changes that might have altered the properties of the enamel surface before sealant application. It may also contain higher proportions of organic material that might hinder the acid penetration into the deeper layers and prevent homogeneous etching of the enamel, hampering resin impregnation and resulting in shorter resin tags [29]. According to Oulis et al. [30], children with a high baseline caries risk showed lower sealant retention rates and higher occlusal caries prevalence following fissure sealants loss compared to those of moderate and low-risk status. The concept of risk-based sealant application can form the basis of a rationale for and effects of sealant placement and specific considerations like tooth morphology, caries history, fluoride history and oral hygiene can be assessed by an experienced clinician for the indication of sealant placement [31, 32]. In the included studies, the study by Durmus et al. was conducted in subjects with high caries risk [22], while patients in the other studies had low caries risk. All individual caries risk was determined at the initial visit with simple indicators and was not reported at the follow-up visits, so changes and the effect of any changes were unclear. Under these circumstances, split-mouth designs may be recommended. Karaman [24] used a parallel design, while others adopted a split-mouth design. In addition, the study by Shindova et al. adopted a parallel design for the subjective parameters of dental anxiety measurement which did not eliminate the effect of individual variation on the surveyed outcomes, affecting the accuracy of the conclusion.

Second is the choice of tooth location. According to a systematic review and meta-analysis from Papageorgiou and previous studies, premolars had better sealant retention rates than that of the first permanent molars. The number of sealants, the complexity of the access to the tooth surface and isolation and variations in the morphology and microscopic structure of the enamel may contribute to these outcomes [33]. In half of the studies that recorded retention rates, sealants were placed on the first permanent molars [22, 23], while the others were placed on permanent premolars and molars [24, 25]. Karaman [24] found no statistical differences between the retention rates of the premolars and molars, while Walsh [25] showed that all the sealants lost were on molars. Location of the loss may be a factor affecting retention rates.

Third is the single evaluation criterion for patient acceptance. Patient acceptance and dental anxiety are subjective perceptions. Thus, rating scales were formulated to quantify their perceptions. Kumur et al. used a visual analogue scale in which pediatric patients were asked to mark their discomfort on a 100 mm line with extreme ends denoting no discomfort to worst possible discomfort during the procedure. According to Naegeli et al. [34], verbal rating scales, visual analogue scales, numeric rating scales, and graphical scales can all be reliable and valid response options in pediatric populations. However, the current empirical basis was insufficient to draw firm conclusions and to make differentiated recommendations. In children between the ages of six and 18, these instruments showed various outcomes. Therefore, diversified evaluations related to subjective parameters should be considered.

Lastly, the lack of comparison between lasers and other preparation methods, the comparison of different kinds of lasers to other chemical or mechanical preparation methods, such as enameloplasty and air-polishing systems, and research on primary molars was a shortcoming of this review. Enameloplasty with burs (fissurotomy shallow taper fissure (STF) and narrow taper fissure (NTF) burs, small one-quarter round burs) conducted with a high- or slow-speed handpiece, has been demonstrated in several in vivo and in vitro studies as a simple, cost-effective and easily accessible method resulting in acceptable sealant retention and reduced microleakage [35–41]. The air abrasion system uses a high-speed stream of purified aluminum oxide particles or bioactive glass delivered by air pressure and has been reported to be an effective method to prepare enamel before the application of the sealant in vitro*,* which needs more research to confirm its clinical effects [42–44].

### Effects of laser parameters on outcome

In our study, we regarded lasers as a whole to analyze the effect of this kind of intervention. As Karaman speculated, different outputs and experimental designs might result in conflicting findings [24]. Subgroup analysis could not be performed, which means that the effects of laser parameters on outcomes are still unclear.

Therefore, the choice of laser parameters in clinical studies has usually referred to those used in previous in vitro studies. For example, Walsh et al. [25] stated that they based the carbon dioxide laser dosimetry on laboratory studies of enamel surface changes and bond strength, as well as on data for pulpal safety. These kinds of choices might also affect the clinical effects of laser preparation. The study by Dumurs et al. [22] used Er: YAG laser at a wavelength of 2.94 μm, 2 W power, 120 mJ energy output and 10 Hz frequency. Mahtab et al. [45] found no difference in the proportion of microleakage between conventional acid etching and Er: YAG laser surface pretreatment before sealant resin was applied to teeth with fluorosis, where the parameters were 30 mJ, 20 Hz, 6 W initially and 120 mJ, 10 Hz, and 1 W on the enamel margins. Unal et al. [46] evaluated the Er:YAG laser as an alternative to acid etching for the application of fissure sealants in pediatric dentistry and found that the Er: YAG laser with 4 W power and 200 mJ energy output resulted in higher microtensile bond strength than the 2 W, 100 mJ Er: YAG laser in permanent teeth. However, in a study of primary teeth, Unal et al. [47] reported that higher microleakage values were found in groups with Er: YAG laser etching (3.25 W, 150 mJ, 25 Hz and 5 W and 20 mJ, 25 Hz) compared to conventional acid-etching. Microleakage in the 3.25 W group and the 5 W group was not significantly different. Kumar et al. [23] used a lower output (1.5 W) to etch enamel, per Berks and colleagues, and observed that Er, Cr: YSGG laser irradiations using 1 W, 1.5 W or 2 W produced etching patterns similar to those produced by etching with acid in the scanning electron microscope (SEM) study. While Vijayan et al. [48] found that the enamel surfaces etched by Er, Cr: YSGG laser at 1.5 W/20 Hz, 2 W/10 Hz and 2 W/20 Hz, 2 W/20 Hz provided the best results with consistency in tag lengths and other studies found that it provided adequate bond strength. Moreover, in vitro studies have shown that the same parameters could result in different outcomes and different parameters could result in similar outcomes. So, while in vitro studies can predict clinical outcomes, real-life performance should be evaluated by clinical studies. More elaborately designed laser dosimetry studies should be conducted in future research.

It should be noted that the use of laser preparation involves some difficulties for the operator. Clinicians must use the correct angle and tip during preparation. A smaller tip may be useful to reduce errors but are associated with problems, such as the need to ensure an equal level of ablation at the same speed, which requires more time and energy compared to larger tips [14]. Thus, theoretical parameters do not always represent actual emissions that impact clinical effects in patients.

### Laser combined with acid preparation

One of our included studies evaluated the clinical effects of Er:YAG laser as an adjunct to acid preparation, showing that it was superior to acid preparation at the end of 18 months. The retention rate for fissure sealants in the laser plus acid group was significantly higher than that in the acid-etched group at 12 (*P* = 0.0161) and 18 (*P* = 0.0227) months. These results agreed with an in vivo study in which Brugnera and colleagues [49] demonstrated that the retention rate of sealants applied after carbon dioxide laser plus acid-etching was significantly better than that of acid-etching preparation. However, the latter study was excluded from this review because it did not describe the randomization process. In the Durmus study, the incidence of caries in the acid-etched group was 22% (*n* = 18) versus 10% (*n* = 8) in the laser plus acid group at 18 months. The difference in caries development between the groups was not significant (*P* > 0.05). In vitro studies showed no statistical difference between the acid and laser combined method and the acid enamel conditioning method [18, 50]. From this perspective, laser combined with acid preparation was probably superior to acid preparation. More high-quality studies should be conducted to confirm this conclusion.

The economic aspect of sealant application should also be considered. Sealant application has to remain simple, rapid and affordable to be used as a prophylactic measure. Although laser combined with acid etching improved the retention of sealants compared to acid etching alone, the cost-benefit ratio of the extra time and cost of the equipment and materials required may not add value [14].

Considering the limited evidence in the present studies, more clinical research is needed to confirm the role of laser preparation in pit-and-fissure sealants in the future. They should be of high methodological quality, design and conduct. Comparison of retention rates, and the incidence of caries, as well as consideration of caries risk, the economic impact and the appropriate choice of study subjects and their psychological evaluation, between laser preparation and other chemical or mechanical preparation methods may further our understanding of the role of lasers as an enamel conditioning procedure before sealant placement.

## Conclusion

In summary, our meta-analysis and systematic review demonstrated that laser preparation was a safe, effective and highly-acceptable method of enamel preparation before sealant placement. The retention rate of pit-and-fissure sealants after laser preparation alone was comparable to that of acid-etching preparation. Furthermore, laser preparation used as a supplementary method to conventional acid-etching enhanced the retention rate of sealants. However, the current study exhibited an overall high risk of bias. Further research with a better study design is required to provide more reliable evidence for clinical application.

## Data Availability

All data generated and analyzed in this study are included within the article or available from the authors.

## Abbreviations

Cl: Confidence interval
Er, Cr: YSGG: Erbium, chromium: yttrium-scandium-galium-garnet
Er: YAG: Erbium: yttrium-aluminum-garnet laser
I^2^: Higgins index
ISRCTN: International Standard Randomised Controlled Trial Number
NTF: Narrow taper fissure
OR: Odds ratio
PRISMA: Preferred Reporting Items for Systematic reviews and Meta-Analyses
STF: fissurotomy shallow taper fissure
